# PBRM1 regulates proliferation and the cell cycle in renal cell carcinoma through a chemokine/chemokine receptor interaction pathway

**DOI:** 10.1371/journal.pone.0180862

**Published:** 2017-08-28

**Authors:** HongKai Wang, YuanYuan Qu, Bo Dai, Yao Zhu, GuoHai Shi, YiPing Zhu, YiJun Shen, HaiLiang Zhang, DingWei Ye

**Affiliations:** 1 Department of Urology, Shanghai Cancer Center, Fudan University, Shanghai, China; 2 Department of Oncology, Shanghai Medical College, Fudan University, Shanghai, China; University of South Alabama Mitchell Cancer Institute, UNITED STATES

## Abstract

*PBRM1* is a novel tumor suppressor gene that can inhibit cancer cell proliferation and predict the outcome of renal cell carcinoma (RCC), but its biological role needs further elucidation. We examined expression of the *PBRM1* gene in RCC cell lines and the effect of PBRM1 on cell proliferation and cell cycle in RCC ACHN cells. Microarray processing and analysis was used to explore novel pathways involved in tumorigenesis related to *PBRM1* knockdown. PBRM1 was expressed at high levels in RCC ACHN cells and lentivirus-mediated *PBRM1* knockdown in these cells caused an increase in the proportion of cells in S phase of the cell cycle and promoted in vitro proliferation and migration. In vivo experiments showed that downregulation of *PBRM1* promoted tumorigenesis in nude mice. In pathway gene chip analysis, the chemokine/chemokine receptor interaction pathway showed the greatest difference in gene expression upon *PBRM1* knockdown. Protein levels of IL6ST and CCL2 were increased, whereas levels of interleukin (IL)-8, IL-6, and CXCL2 were decreased, in knockdown cells. Re-expression of IL-8 in *PBRM1* knockdown ACHN cells could significantly decrease cell proliferation/migration and induced cell arrest in the G2/M phase. These findings indicate that PBRM1 alters cell cycle progression and inhibits proliferation and migration of ACHN cells through the chemokine/chemokine receptor pathway.

## Introduction

Renal cell carcinoma (RCC) is the most common type of cancer in the kidney and accounts for approximately 3% of all adult malignancies[1]. Among RCCs, clear cell RCC (ccRCC) is the most common subtype, accounting for approximately 70%–75% of cases[2], and is more likely to present with advanced T stage, metastatic disease, and higher grade[3]. Alteration in the von Hippel-Lindau (*VHL*) gene is the hallmark of ccRCC; however, inactivation of VHL has not been found to consistently correlate with prognostic features of ccRCC[4]. Recently, exome sequencing has unveiled additional genes that are mutated in ccRCC, including *PBRM1*, *BAP1*, and *SETD2*[5]. As the second most frequently mutated gene after *VHL*, the role of PBRM1 in ccRCC tumorigenesis is of great interest.

PBRM1 encodes the BAF180 protein, which is a subunit of the ATP-dependent chromatin remodeling complex called SWI/SNF (SWItch/Sucrose NonFermentable). Mutations in SWI/SNF, and the subsequent abnormal function of SWI/SNF complexes, are among the most frequent gene alterations in cancer[6]. In ccRCC, the majority of *PBRM1* mutations lead to loss of the protein[7]. Clinical data indicated that negative expression of PBRM1 is correlated with advanced tumor stage, low differentiation grade, and worse patient outcome[8,9]. However, the biological role of PBRM1 and the molecular pathways through which downregulation of PBRM1 promotes the growth of RCC needs further elucidation.

In this study we investigate the expression and function of PBRM1 in ccRCC cells in vitro and in vivo, and present data suggesting that PBRM1 may be a regulator of chemokine/chemokine receptor pathways.

## Results

### Downregulation of PBRM1 in RCC ACHN cells using lentivirus

**Fig 1:**
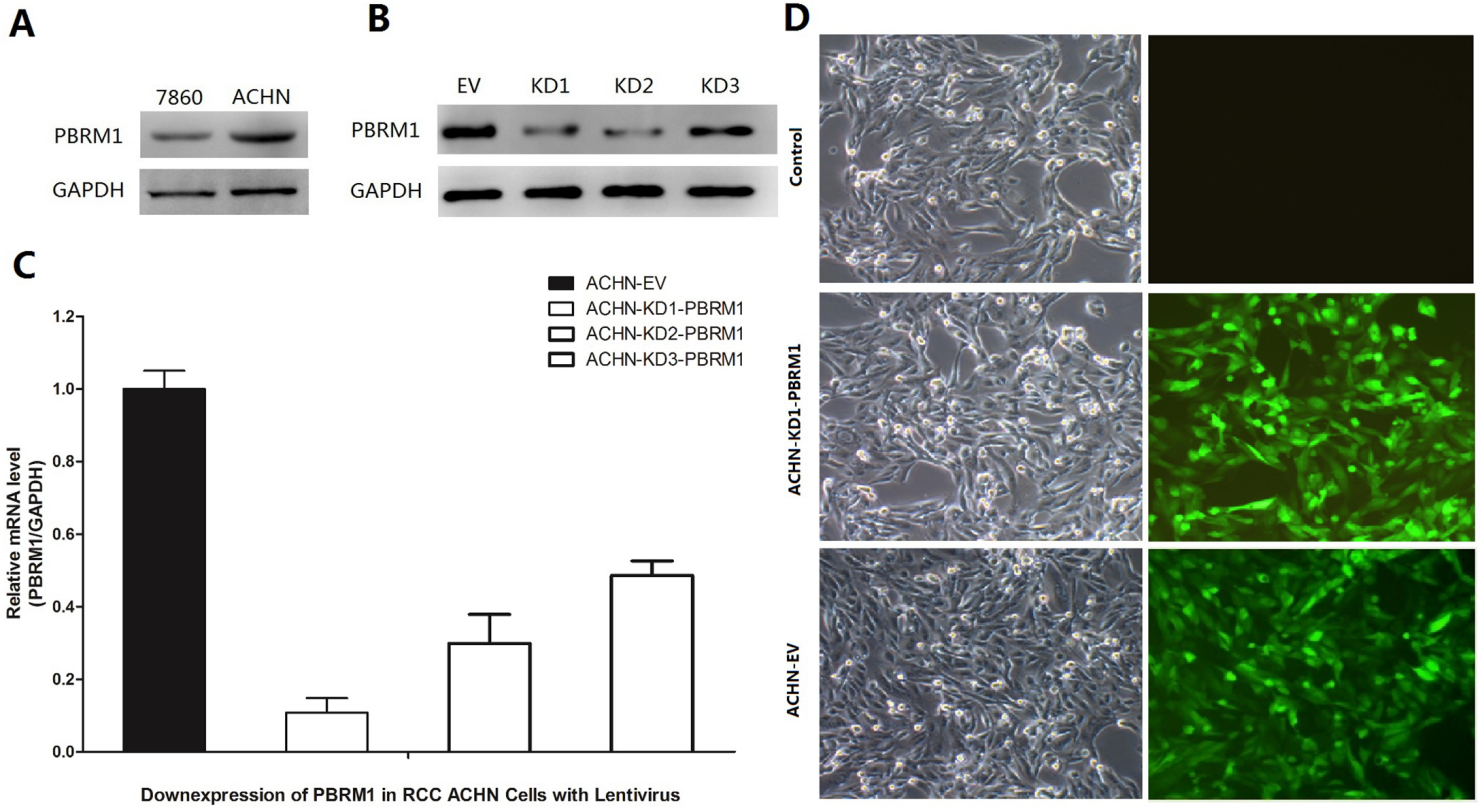
PBRM1 knockdown ACHN cells showed favorable infection efficiency. (A) Expression levels of PBRM1 were relatively high in the metastatic RCC cell line ACHN compared with the primary RCC cell line, 786–0. (B, C, D) Downregulation of PBRM1 in RCC ACHN cells using lentivirus.

Western blot analysis was performed to detect PBRM1 expression in the RCC cell lines ACHN and 786–0. As shown in Fig 1A, the levels of PBRM1 expression were relatively high in the metastatic RCC cell line ACHN. We knocked down *PBRM1* in ACHN RCC cells using three different PBRM1 RNAi sequences to study the biological functions of PBRM1. ACHN cells were transfected with virus containing PBRM1 RNAi (KD1,2,3-PBRM1) or empty virus (EV) and performed RT-PCR and western blotting to detect PBRM1 expression after transfection. As shown in Fig 1B and 1C, the PBRM1 level was significantly lower in ACHN-KD1-PBRM1 compared with ACHN-EV. The infection efficiency was nearly 100% (Fig 1D).

### PBRM1 silencing promoted cell proliferation and migration/invasion ability and significantly increased the S phase population of ACHN cells

**Fig 2:**
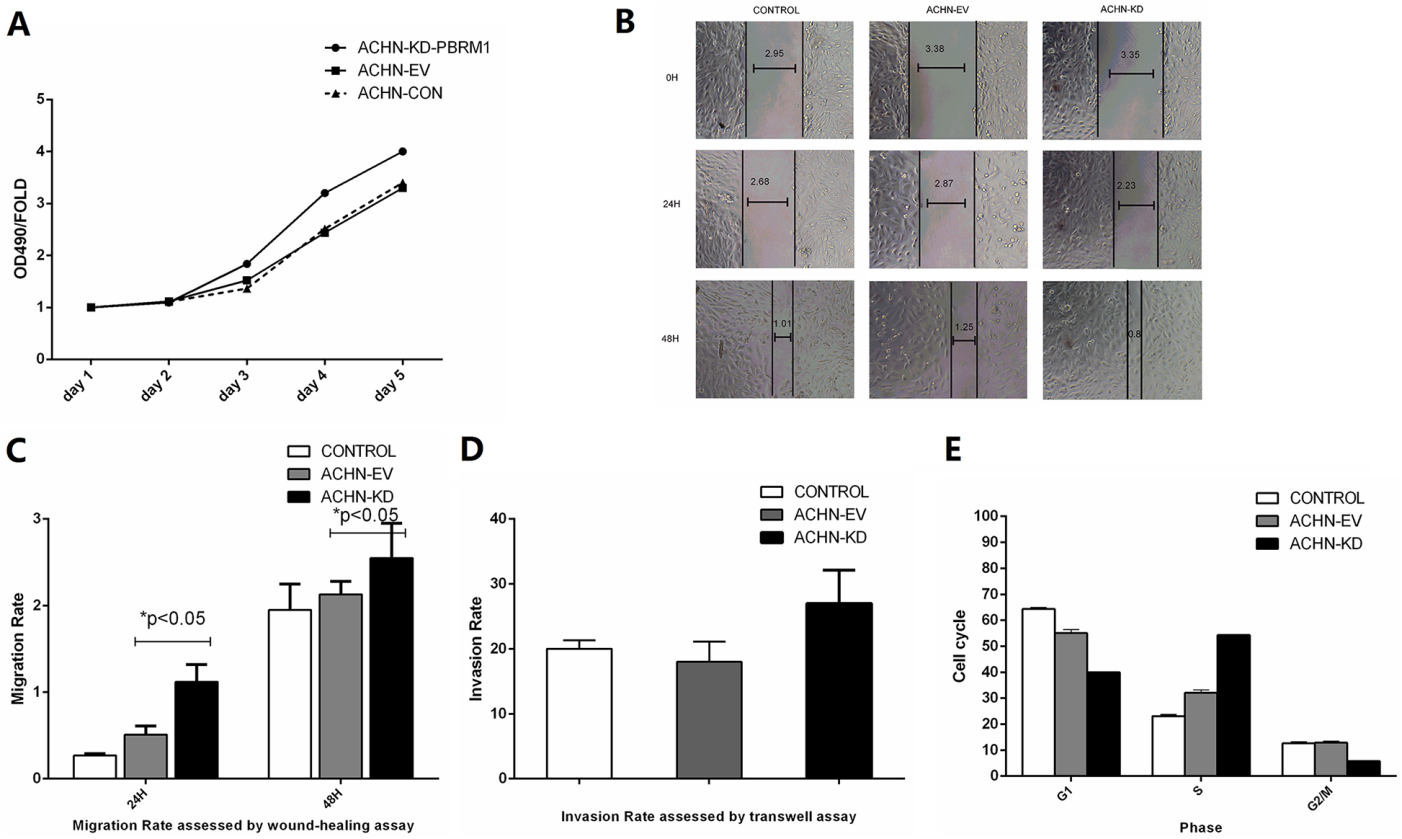
PBRM1 silencing regulates tumorigenic prosperities. (A) Proliferation capability of stable transfected cell lines by MTT assay. (B, C, D) Wound-healing and Transwell cell invasion assays were used to examine migration and invasion abilities of ACHN-KD-PBRM1 cells. (E) Cell cycle alterations of stable PBRM1 knockdown cells were detected by flow cytometry.

The growth curve determined from an MTT assay showed that PBRM1 silencing increased the proliferation rate compared with transfection with empty virus (P < 0.05, Fig 2A). Wound-healing and Transwell cell invasion assays showed that the migration and invasion abilities of ACHN-KD-PBRM1 were stronger than those of ACHN-EV (Fig 2B, 2C and 2D). *PBRM1* knockdown ACHN cells exhibited fewer cells in G1 phase and more cells in S phase (Fig 2E). These results indicate that artificial reduction of PBRM1 expression promotes the proliferation of RCC cancer cells, suggesting that PBRM1 may play an important role in the progression of renal cancer.

### Downregulation of PBRM1 promoted tumorigenesis in nude mice

**Fig 3:**
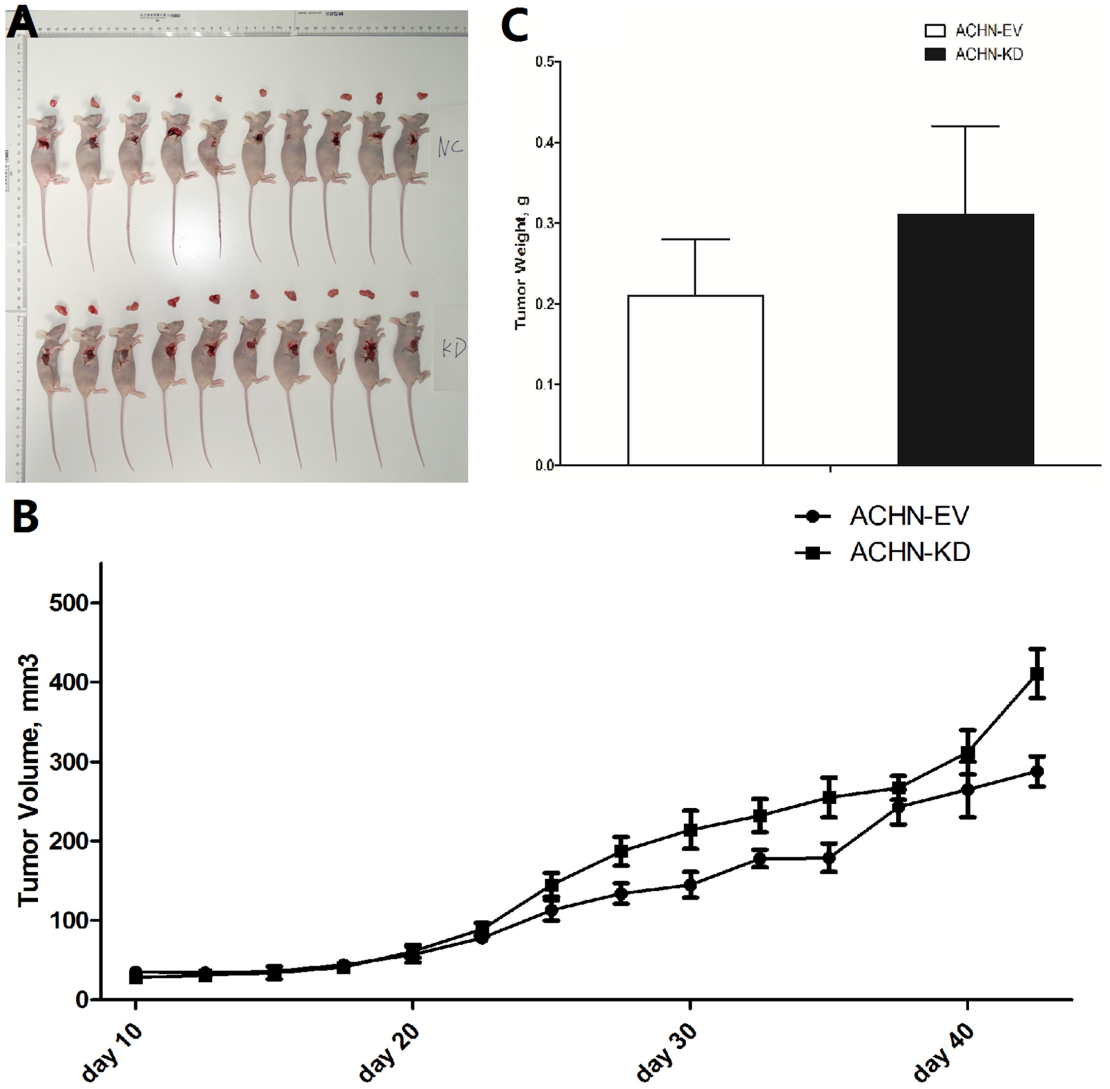
Downregulation of PBRM1 promoted tumorigenesis in nude mice. (A) A xenograft tumor model was established. (B) The volume of the PBRM1-knockdown tumors was larger than that of the mock-transfected tumors. (C) Tumors harvested from ACHN-KD-PBRM1 treated mice were larger than those of the mock-treated mice on day 45, p<0.05.

To determine whether PBRM1 expression is correlated with tumorigenesis *in vivo*, we established a xenograft tumor model (Fig 3A). After subcutaneous injection of nude mice with ACHN-KD-PBRM1 and ACHN-EV, tumor volume was measured with a Vernier caliper twice a week. The volume of the PBRM1-knockdown tumors was larger than that of the mock-transfected tumors (Fig 3B). Tumors harvested from ACHN-KD-PBRM1 treated mice were larger than those from the mock-treated mice on day 45, p<0.05 (Fig 3C).

### Pathway gene chip analysis revealed that PBRM1 knockdown alters the chemokine/chemokine receptor interaction pathway

**Fig 4:**
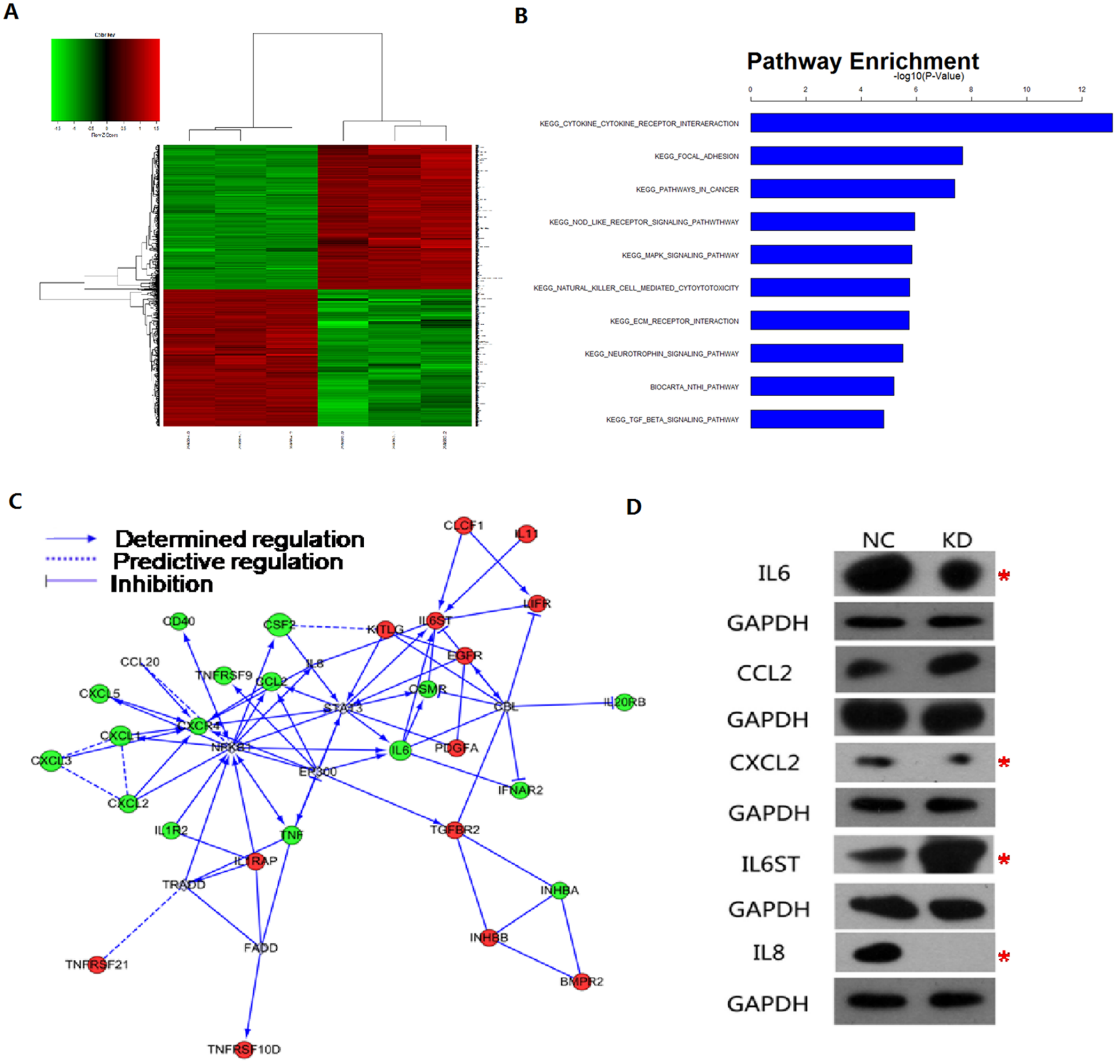
Pathway gene chip analysis after PBRM1 inhibition. (A) Gene expression profiling using the Affymetrix Human Gene 1.0 ST platform identified 872 transcripts that were significantly differentially expressed(S1 File). (B) Pathways analysis revealed that chemokine/chemokine receptor interaction was the top modulated canonical pathway following *PBRM1* knockdown (p<10^−12^). (C) A gene co-expression network was generated according to the differentially expressed genes. (D) Western blot analysis confirmed increased protein levels of IL-6ST and CCL2, whereas the protein levels of IL-8, IL-6, and CXCL2 were decreased.

To gain insights into the mechanisms of PBRM1 function we compared the transcriptomes of cells transfected with KD-PBRM1 or EV. Gene expression profiling using the Affymetrix Human Gene 1.0 ST platform identified 872 transcripts that were significantly differentially expressed based on a p<0.05 threshold (Fig 4A). Functional analysis based on the KEGG pathway database revealed that *PBRM1* knockdown modulated key pathways, in particular cytokine/cytokine receptor interaction, focal adhesion, pathways in cancer, NOD-like receptor signaling pathway, and MAPK signaling pathway (Fig 4B). Pathway analysis revealed that chemokine/chemokine receptor interaction was the top modulated canonical pathway following *PBRM1* knockdown (p<10^−12^) (Fig 4B). A gene co-expression network was generated according to the differentially expressed genes (Fig 4C). Genes that showed significantly altered expression are shown in Table 1. Based on a combination of statistical criteria and pathways analysis, we validated the protein expression of several significant pathway-associated genes and confirmed increased protein levels of interleukin (IL)-6ST and CCL2 and decreased protein levels of IL-8, IL-6, and CXCL2 after *PBRM1* knockdown (Fig 4D).

**Table 1:** Gene expression profiling of significantly changed genes.

Gene Symbol	Regulation	Fold Change
CCL20	down	49.40524
IL8	down	48.246643
CSF2	down	9.922694
CXCL3	down	6.9511065
CXCL1	down	5.9785233
IL6	down	5.6783504
CCL2	down	4.0504875
CXCL5	down	3.9426293
TNF	down	3.4657867
CXCL2	down	3.139261
IL11	up	2.1686192
PDGFA	up	1.8415861
CLCF1	up	1.8080981
EGFR	up	1.7747135
IL1R2	down	1.7700913
IL20RB	down	1.7270195
CXCR4	down	1.6770942
IL6ST	up	1.6687354

### IL-8 re-expression in KD-PBRM1 cells resulted in decreased proliferation and migration and cell arrest in G2/M phase

**Fig 5:**
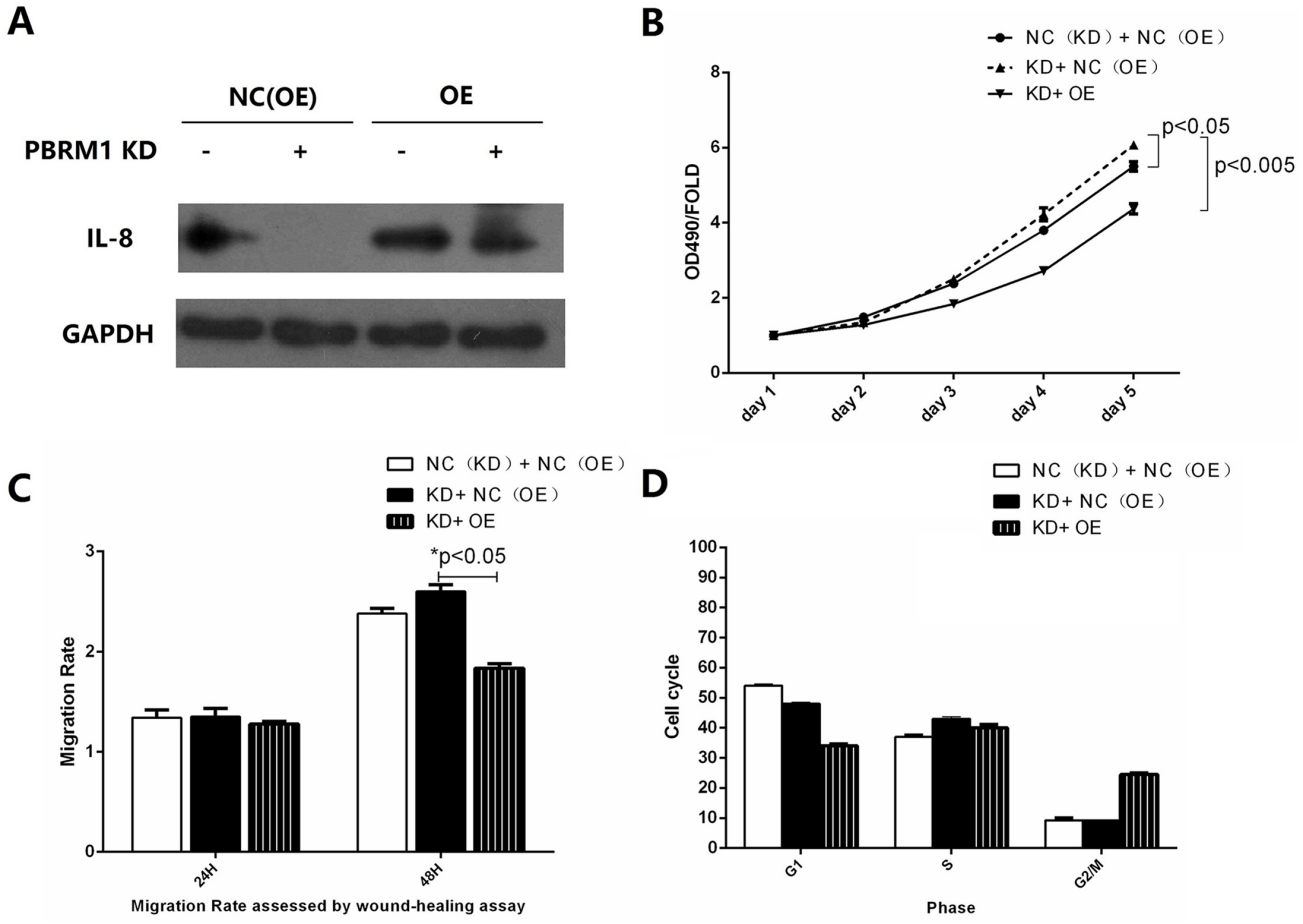
Overproliferation state of KD-PBRM1 cells could be reversed by re-expression of IL-8. PBRM1 re-expression in KD-PBRM1 cells showed favorable infection efficiency. (B) Re-expression of IL-8 decreased the proliferation rate. (C) Re-expression of IL-8 decreased the migration ability. (D) Re-expressed IL-8 cell caused cell arrest in G2/M phase.

PBRM1 re-expression in KD-PBRM1 cells showed favorable infection efficiency(Fig 5A). The growth curves determined from MTT assays showed that re-expression of IL-8 decreased the proliferation rate and migration ability of OE+KD-PBRM1 cells compared with KD-PBRM1 cells (P < 0.05, Fig 5B and 5C). KD-PBRM1 ACHN cells that re-expressed IL-8 exhibited fewer cells in G1 phase and S phase, and more cells in G2/M phase (Fig 5D). These results show that the over proliferation state of KD-PBRM1 cells could be reversed by re-expression of IL-8, suggesting that the chemokine/chemokine receptor pathway might play an important role in the progression of renal cancer.

## Discussion

Clear cell renal cell carcinoma accounts for 70%–80% of all kidney cancers, and is known to exhibit very frequent inactivation of the von Hippel-Lindau gene (*VHL*) as a result of either somatic mutations or epigenetic alterations[10]. In addition to *VHL*, sequencing studies have revealed that truncating mutations in *PBRM1*, which encodes a subunit of the ATP-dependent chromatin remodeling complex of SWI/SNF, are present in more than 40% of ccRCCs[5]. Other frequently mutated genes in ccRCC are *SETD2*, *TCEB1*, *BAP1*, and *KDM5C*[11]. It is interesting that *PBRM1*, *BAP1*, and *SETD2* are all located at chromosome 3p, close to the 3p25 locus, indicating that these tumor suppressors might be functionally linked. As *PBRM1* is the second most frequently mutated gene after *VHL*, its interaction with other mutated genes and its role in ccRCC tumorigenesis and progression are areas of great interest.

In the current study we examined expression of the *PBRM1* gene in RCC cell lines and demonstrated that lentivirus-mediated *PBRM1* knockdown in ACHN cells induces cell proliferation, migration, and invasion. Silencing of *PBRM1* also resulted in an increase in the number of cells in S phase of the cell cycle. In a mouse model of renal cell carcinoma, PBRM1 influenced the growth of tumors in nude mice subcutaneously injected with ACHN cells. Finally, pathway gene chip analysis revealed that *PBRM1* knockdown predominantly alters the cytokine/cytokine receptor interaction pathway. Increased protein levels of IL-6ST, and decreased levels of IL-8, IL-6, and CXCL2, were observed following *PBRM1* knockdown. Finally, we showed that the overproliferation state of KD-PBRM1 cells could be reversed by re-expression of IL-8. Above all, we identified a critical role for the chemokine/chemokine receptor pathway in PBRM1-induced growth inhibition in RCC. This result enhances our understanding of PBRM1-induced tumorigenesis.

Previous in vitro and in vivo studies revealed other biological functions of PBRM1. *PBRM1* deletion was shown to cause a deficiency in mice leading to embryonic lethality[12], and may also enhance Th2 differentiation and increase IL-10 expression[13]. Other studies reported that PBRM1 is required for cohesion and prevention of genomic instability[14], and is important for DNA double-strand break–induced transcriptional silencing and promotes repair of a subset of DNA damage[15]. PBRM1 was shown to be a critical transcriptional regulator of p21 during tumorigenesis in breast cancer[16], and also regulates p53 function by influencing p53 transcriptional activity and is required for p53-induced replicative senescence[17]. A recent study indicated that *PBRM1* knockdown leads to dysregulation of chromosomal instability and cellular proliferation, indicating that the loss of PBRM1 in RCC may give rise to a chromosomal instability/spindle checkpoint expression phenotype. Notably, Chowdhury et al. re-expressed PBRM1 in the Caki2 RCC cell line and observed upregulation of chemokine receptors such as CCL20 (0.46-fold) and CXCL5 (0.61-fold). Clinical data indicate that PBRM1 mutation can also lead to alterations in chemotaxis[18].

How PBRM1 influences the expression of chemokines and their receptors is of great interest; however, to date only indirect linkages have been found. A study of Th2 cells suggested that PBRM1 might function as a repressor of IL-10 by binding directly to regulatory elements in the *IL10* locus. In the absence of PBRM1, enhanced Th2 differentiation and IL-10 expression was observed, and BAF recruitment and histone acetylation at the *IL10* locus was increased^13^. IL-10 may inhibit chemokine expression and neutrophil accumulation through mRNA destabilization and NF-kappaB inhibition, in addition to polymorphonuclear leukocyte (PMN)–derived chemokine expression[19]. In another study, Jeong et al. found that the SWI/SNF chromatin-remodeling complex modulates peripheral T-cell activation and proliferation by controlling activator protein-1 (AP-1) expression[20], and AP-1 may modulate the expression of certain chemokines such as IL-8[21] and CXCL2[22]. Further investigations should be performed to elucidate the association between PBRM1 and chemokine pathways.

Chemokines are small secreted proteins that function in leukocyte recruitment to inflammatory sites and secondary lymphoid organs[23]. They have been classified into four main subfamilies: CXC, CC, CX3C, and XC. All of these proteins exert their biological effects by interacting with G protein-linked transmembrane receptors called chemokine receptors[24]. Research has shown that they have direct impacts on the biology of RCC by promoting angiogenesis and metastasis or by activating the hypoxia-inducible factor (HIF)-α pathway. CXC chemokines that contain the ELR motif (ELR-CXC chemokines) were found to be potent angiogenic factors through CXCR2[25], whereas members that lack the ELR motif inhibit angiogenesis through CXCR3[26]. CXCR2 and CXCR3 and their ligands are highly expressed in RCC tumors[27,28] and may be associated with a favorable prognosis of RCC[29]. CXCR4, another chemokine receptor, functions in a major mechanism for RCC metastasis via interaction with its ligand CXCL12[30]. Other studies showed that CXCR4 is associated with the HIF pathway in RCC. Staller and colleagues found that the von Hippel-Lindau tumor suppressor protein pVHL negatively regulates CXCR4 expression through its capacity to target HIF for degradation under normoxic conditions. ccRCC patients with *VHL* gene mutations revealed an association between strong CXCR4 expression and poor tumor-specific survival[31]. There is a lack of articles discussing CCL2 and CXCL2 in the context of tumorigenesis of RCC; however, these chemokines were found to be associated with tumor growth and progression of melanoma and breast cancer[32,33]. Chemokines may also induce resistance to targeted therapy. Huang and colleagues observed an increase in the secretion of IL-8 in sunitinib-resistant tumors, and coadministration of IL-8 neutralizing antibodies resulted in resensitization to sunitinib[34]. Understanding the mechanisms of chemokine-induced tumorigenesis may provide novel insights into RCC, which may in turn lead to improved therapies.

Our experimental study presented some limitations. First, experiments were mainly performed using the ACHN cell line, and more cell lines should be used in further investigations to establish a comprehensive conclusion. Second, we did not demonstrate how PBRM1 acts on the chemokine/chemokine receptor pathway. Further research is needed to provide insights into the mechanism underlying the tumor-suppressive action of PBRM1 and the chemokine/chemokine receptor pathways.

## Conclusion

PBRM1 may alter cell cycle progression and inhibit proliferation and invasion of ACHN cells through the chemokine/chemokine receptor pathway. Understanding the contribution of PBRM1 dysregulation and its associated pathways to clinical disease progression and outcome are important future areas of renal cancer research.

## Methods

All experimental protocols were approved by the Review Committee for the Use of Human or Animal Subjects of Fudan University and the experimental methods were performed in accordance with relevant guidelines and regulations.

### Cell culture and lentivirus transduction

Human RCC ACHN cells were cultured in Eagle’s minimum essential medium (MEM) supplemented with 10% fetal bovine serum (FBS) (Hyclone, Logan, UT, USA) at 37°C under 5% CO_2_. To construct the KD-PBRM1 vector, the coding sequence of the PBRM1 RNAi was inserted into pGC-LV-GFP vector (Genechem, Shanghai). Lentiviral particles were produced in HEK293T cells by co-transfection of KD-PBRM1 or mock vector with psPAX2 and pMD2.G packaging vectors. The ACHN cells were infected at a multiplicity of infection (MOI) of 5 with either lentivirus containing the PBRM1 RNAi (KD-PBRM1) or empty virus (EV) with 6 μg/mL of polybrene according to the manufacturer’s instructions. Western blot analysis was performed to examine protein expression in ACHN cells infected with KD-PBRM1. The following primers were used for RT-PCR: PBRM1 sense 5´-AGCTGCTGCACGCTATGAAG-3´, PBRM1 antisense 5´-CCTTGGTTATTCCGACAACTCC-3´, GAPDH sense 5´- TGACTTCAACAGCGACACCCA-3´, and GAPDH antisense 5´- CACCCTGTTGCTGTAGCCAAA-3´. The KD-PBRM1 cells were infected with lentivirus LVKL7166-3 containing CXCL8 overexpression vector (KD+OE-PBRM1 cells) and IL-8 expression was examined by western blotting.

### Cell proliferation assay

To examine the effect of PBRM1 on cell growth, ACHN cells were infected with lentivirus containing either the *PBRM1* gene (KD-PBRM1) or empty virus (EV). The infected cells were seeded into 96-well plates and incubated for 1 to 5 days. Subsequently, 20 μL of 3-(4,5-dimethylthiazol-2-yl)-2,5-diphenyl tetrazolium bromide (MTT, Sigma) solution (5 mg/mL, Sigma) was added to each well, and the plates were further incubated for 3 h. Crystals were dissolved in 0.04 M HCl in isopropanol and the absorbance at 490 nm was measured with a microplate reader (Bio Rad, Hercules, CA, USA). The experiments were independently repeated three times.

### Western blot analysis

Cell extracts were obtained using lysis buffer containing 5 mmol/L EDTA, 1 mmol/L phenylmethylsulfonyl fluoride, 1 mmol/L dithiothreitol, 0.1 mmol/L leupeptin, 75 μmol/L pepstatin A, 150 mmol/L NaCl, and 0.1% Triton X-100. The lysate was centrifuged for 30 min (15,000 rpm, 4°C) and the supernatant was collected for western blot analysis. Samples (20 μg) were electroblotted onto nitrocellulose membranes. Antibodies against human PBRM1, IL-6, IL6ST, IL-8, CXCL2, CCL2, and GAPDH (Abcam) were used as the primary antibodies and peroxidase-conjugated goat anti-rabbit or anti-mouse antibodies (Santa Cruz Biotechnology) were used as the secondary antibodies. Protein bands were visualized using the SuperSignal West Pico Chemiluminescent Substrate (Pierce).

### Cell cycle analysis

Cells grown in regular growth media or serum-free media for 36 h were collected, fixed in methanol, and stained with PBS containing 10 μg/mL propidium iodide and 0.5 mg/mL RNase A for 15 min at 37°C. The DNA content of the labeled cells was measured using the FACSCalibur flow cytometry system (BD Biosciences). Each experiment was performed in triplicate.

### Migration and invasion assays

Qualitative assessment of cell migration was conducted by the wound-healing assay in which a monolayer of cells was scratched with a 200-μL pipette tip and the wound was monitored for closure. Transwell cell invasion was quantified by seeding cells (8×10^4^ cells) in serum-free medium onto the top layer of 24-well BD BioCoat 8.0-mm PET membrane inserts (BD Biosciences). After 48 h, migrating or invading cells were washed with PBS, fixed with 10% formalin, stained with 0.5% crystal violet, and counted using bright-field microscopy. All conditions were conducted with three replicates.

### In vivo tumorigenesis study

All experimental protocols were approved by the Review Committee for the Use of Human or Animal Subjects of Fudan University and experimental methods were performed in accordance with relevant guidelines and regulations. Housing and husbandry was according to the standard guideline and cleaning was performed every 3 days. If the drugs or tumors induced significant illness, euthanasia was performed by 2% nembutal injection. For power analysis calculation, the principle is to reduce the number of mice required without influencing the statistical significance. For this research we needed at least six mice for each group. To account for unexpected death we increased this number to 10. ACHN-KD-PBRM1 and ACHN-EV cells were collected and injected subcutaneously into the right and left flanks (5 × 10^6^ cells/site) of nude mice (10 mice/group). To monitor tumor growth, the tumor was measured using Vernier calipers and volume was calculated according to the formula volume = W2 × *L* × 0.5 (where W and L represent the largest and second largest tumor diameters [cm]) and then plotted. Mice were humanely sacrificed on day 45, and the tumors were weighed and photographed.

### Microarray processing and analysis

Total RNA was isolated from ACHN-KD-PBRM1 and ACHN-EV cells. RNA samples were analyzed by microarray expression profiling using the Affymetrix Human Gene 1.0 ST platform according to the manufacturer’s instructions. A total of 2.5 mg of fragmented and labeled cDNA was generated using the Affymetrix GeneChip WT Terminal Labeling and Controls Kit and hybridized to Human Gene 1.0 ST arrays according to the manufacturer’s instructions (Affymetrix). Arrays were washed, stained, and processed using Affymetrix GeneChip Fluidics Station 450 systems, and then imaged using Affymetrix GeneChip Scanner 3000 7G for subsequent generation of raw data (*CEL files). Genes that were significantly differentially expressed between ACHN-KD-PBRM1 and ACHN-EV were selected on the basis of P value <0.05. Functional analysis based on the KEGG pathway database was performed.

### Statistical analysis

Experiments were repeated three times and the results were expressed as the mean ± standard deviation (SD). Student’s t-test or ANOVA followed by a post hoc test was used to compare the values of the tumor samples with those of the control samples. A value of P <0.05 was considered to be statistically significant.

The study protocol was approved by the Institutional Review Board of our hospital. All methods were carried out in accordance with approved guidelines.

## Supporting information

S1 File Gene expression profiling and pathway analysis. **Link**: https://figshare.com/s/c3310e91026bb2b15e4a.
(XLSX)
